# Bioavailability of Isothiocyanates From Broccoli Sprouts in Protein, Lipid, and Fiber Gels

**DOI:** 10.1002/mnfr.201700837

**Published:** 2018-04-14

**Authors:** Teresa Oliviero, Simone Lamers, Edoardo Capuano, Matthijs Dekker, Ruud Verkerk

**Affiliations:** ^1^ Food Quality and Design Group Department of Agrotechnology and Food Sciences Wageningen University Bornse Weilanden 9 6708 WG Wageningen The Netherlands

**Keywords:** bioavailability, fibers, gels, isothiocyanates, lipids, proteins

## Abstract

**Scope:**

Optimization of bioavailability of dietary bioactive health‐beneficial compounds is as important as increasing their concentration in foods. The aim of this study is to explore the change in bioavailability of isothiocyanates (ITCs) in broccoli sprouts incorporated in protein, fiber, and lipid gels.

**Methods and results:**

Five participants took part in a cross‐over study and collected timed urine samples up to 24 h after consumption of proteins, dietary fibers, and lipid gels containing broccoli sprouts powder. Sulforaphane and iberin metabolites were determined in the urine samples. Samples in which sulforaphane and iberin were preformed by myrosinase led to a higher bioavailability of those compounds. Compared to the control broccoli sprout, incorporation of sprouts in gels led to lower bioavailability for preformed sulforaphane and iberin (although for sulforaphane the lower bioavailability was not significantly different) whereas for the gels rich in their precursors, glucoraphanin and glucoiberin, the opposite trend was observed (although not significantly different).

**Conclusion:**

This explorative study suggests that ITCs bioavailability can be modulated by food structure and composition and further and deeper investigations are needed to develop food products that lead to an optimized ITCs bioavailability.

## 1 Introduction

Many studies have suggested the beneficial effect on health from consumption of *Brassica* vegetables.^1^ These vegetables belong to the Brassicaceae family, also called Cruciferae, including many economically important plants such as leaf and root vegetables, oilseed, and condiment crops. The health effect has been mainly attributed to the occurrence of a class of compounds named glucosinolates (GLs) which are β‐thioglucoside‐*N*‐hydroxysulfates. GLs can be hydrolyzed by the plant endogenous β‐thioglucosidase enzymes (EC3.2.1.147), named myrosinase (MYR). This happens very quickly upon cell damage, such as chewing or grinding. Among the different classes of breakdown products, isothiocyanates (ITCs) are those reported to have prominent health‐beneficial effects.^2^

The health‐beneficial effects of ITCs are the results of a multitude of molecular mechanisms that act simultaneously, which include modulation of xenobiotic metabolism; modulation of inflammation; regulation of apoptosis, cell cycle arrest, angiogenesis and metastasis; regulation of epigenetic events; and reduction of advanced glycation end products.^3^ All these mechanisms contribute to the potential protective effect against chronic diseases by the regular consumption of *Brassica* vegetables. However, the formation of ITCs depends on many factors, including domestic preparation of *Brassica* vegetables. The consumption of raw vegetables leads to the hydrolysis of GLs during chewing. Whereas, in vegetables cooked for a long time, no GL hydrolysis occurs because MYR is inactive.^4^, ^5^, ^6^ The fraction of GLs escaping absorption in the small intestine and reaching the large intestine can be hydrolyzed by means of the MYR‐like activity of the gut microbiome.^6^, ^7^, ^8^ Moreover, it is shown that consumption of just shortly cooked vegetables can be even more health beneficial than the consumption of raw vegetables.^9^ Although a short cooking can partially reduce MYR activity in the vegetables, a reduction of the activity of the plant endogenous epithiospecifier protein can promote the formation of ITCs over other more undesired breakdown products.^9^ Moreover, acidic (pH 4) and alkaline (pH 8) conditions, which can be related to cooking preparations (seasoning), showed to be the optimal pH conditions to obtain higher ITC concentrations and water dilution may favor ITC formation (e.g., making soup) for some Brassicaceae.^10^

The ITCs, either formed orally or in the gut, are metabolized mainly by the mercapturic acid pathway. After an initial conjugation with glutathione, the ITCs are further enzymatically modified to produce cysteinylglycine–, cysteine– and *N*‐acetylcysteine–ITC conjugates, all of which are dithiocarbamates.^11^ By measuring those metabolites in the blood and in the urine, the bioavailability of ITCs can be assessed.^7^, ^8^, ^11^, ^12^

Studies investigating the bioavailability of ITCs in humans after consumption of differently thermally treated *Brassica* vegetables report that ITC bioavailability is proportional to the amount formed from GL hydrolysis.^6^, ^7^, ^8^ A less explored area is the role of other food components on bioavailability of ITCs during consumption of vegetables, as it happens in a regular meal. ITCs can react with free amino groups and sulfhydryl side chains of proteins,^13^ potentially reducing ITC bioaccessibility. On the contrary, an in vivo study showed that after consumption of a whole meal that included meat, the absorption of ITC was between one‐ and fivefold higher compared to ingestion without meat depending on the type of ITC.^14^ This effect was explained by the presence of fat that can increase the absorption of lipophilic compounds through the micellar phase.^15^, ^16^, ^17^ Dietary fiber could also play a role in the bioavailability of ITC, because it can encapsulate the compounds and thereby postponing their absorption or by changing digesta rheology.^18^ A straightforward study, in which the effect of single compounds on bioavailability of ITCs is investigated is needed to develop food products and diets with optimized ITCs bioavailability.

The aim of this study is to explore the effect of protein, dietary fiber, and fat, on the bioavailability of ITC in a human intervention. To minimize the effect of food structure, we have created a food grade model system for the food matrix of dry powder prepared from broccoli sprouts incorporated in mono‐macromolecular gels. Those gels were prepared using one single macromolecular ingredient, namely gelatine, sodium alginate, and olive oil/candelilla wax as representatives of proteins (P), dietary fiber (F), and oil (O), respectively. As vegetable, freeze‐dried broccoli sprouts (BS), were used to ensure higher ratio of GLs or ITCs/vegetable matrix. The BS and the gels were prepared in order to obtain gels either with ITCs already formed (P+, F+, and O+) or with only GLs and no MYR (P−, F−, and O−). Two types of control samples were prepared: BS with already formed ITCs (C+) and BS with GLs and inactivated MYR (C−). Five participants consumed the samples in a cross‐over study design. In this explorative study, we focused on the most abundant GLs present in BS: glucoraphanin (GR), of which ITC is sulforaphane (SR) and glucoiberin (GI), of which ITC is iberin (IB). The SR and IB metabolites were measured in 24 h timed urine samples before and after consumption of the samples. The cumulative excretion, the bioavailability, and the maximum excretion peak times of the SR and IB metabolites were determined.

## 2 Experimental Section

### 2.1 Preparation of Broccoli Sprouts

Broccoli sprouts (BS) were obtained from Koppertcress (Monster, The Netherlands). The BS were cut with stem and leaf, discarding the roots. Half of it was frozen and freeze dried in 3 days. The other half was microwaved at 900 W for 6 min to inactivate the plant endogenous MYR.^7^ Then the microwaved BS were stored overnight at −20 °C and the day after the BS were placed in the freeze dryer. The samples were dried in 3 days. The two batches of freeze‐dried BS were ground (particle size <450 μm) (Retsch MM 400 milling machine). As a result, two batches of BS were obtained, one with active MYR and the other one with MYR inactive.

### 2.2 Preparation of Mono‐Macromolecular Food Matrices

#### 2.2.1 Rehydration of the Broccoli Sprouts Powder

**Figure 1:**
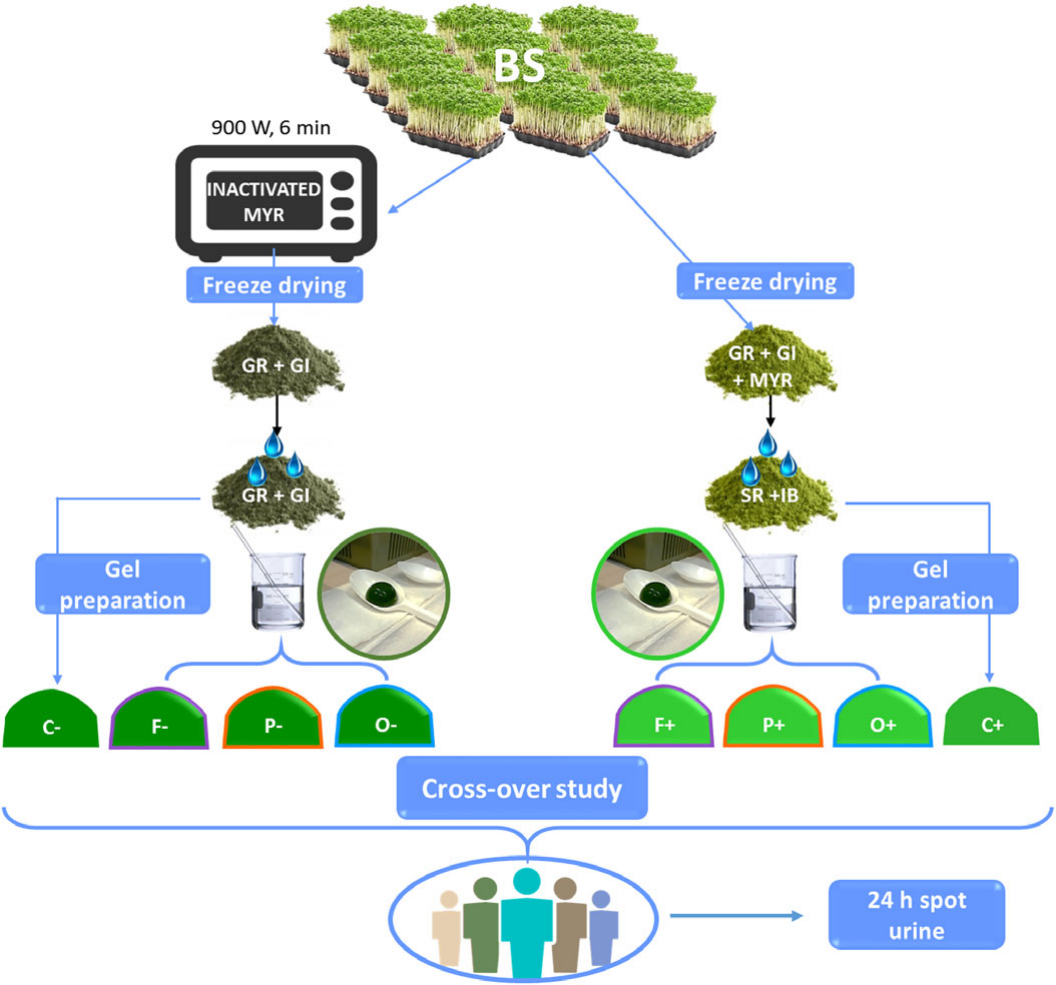
Schematic representation of the sample preparation and the intervention study. Broccoli sprouts (BS), proteins (P), dietary fiber (F), and oil (O). Controls (broccoli sprouts) (C). Myrosinase (MYR), glucoraphanin (GR), glucoiberin (GB), sulforaphane (SR), and iberin (IB). Samples with already formed isothiocyanate (+) and samples with glucosinolates and inactivated myrosinase (−).

The preparation of the mono‐macromolecular gels is illustrated in **Figure** 1. The amounts of BS powder required for the gels was weighed and the required amount of water was added (see **Table** 1), making sure that the powder was rehydrated uniformly. Then the mixture was allowed to stand for 40 min at 40 °C. At the same time the preparation of the mono‐macromolecular gels took place. In case of the control samples, BS with inactivated MYR (BS−) and BS with activated MYR (BS+), the rehydration was performed 40 min before the intervention when the samples were ready to be consumed. The controls after rehydration are called C− and C+.

**Table 1:** Formulations of gel matrices with broccoli sprouts powder (BS) per one gel type, and number of gels served at each session. Controls were BS with glucosinolates and inactivated myrosinase (C−) and BS with already formed isothiocyanates (C+). BS samples were hydrated with the same amount of water (e.g., 0.64 g BS plus 0.64 mL water)

Gel type	Gelling agent	BS per sample [g]	Gel powder [g]	% Gel powder in water or oil	Number of gels served for each session
Fiber (F)	Sodium alginate	0.32	0.22	3.70	2
Protein (P)	Gelatine	0.32	0.62	6	2
Oil (O)	Candelilla wax	0.64	1.34: 1.00	1.34	1
Controls (C)	n.a.	0.64	n.a.	n.a.	1^a^

a The controls samples were not mixed with gel.

n.a., not applicable.

#### 2.2.2 Matrix and Gelling Procedure

The macromolecule material was prepared freshly every session in weight ratios as shown in Table 1. The fiber (sodium alginate, 9005‐38‐3, Sigma, Aldrich) and gelatine (9000‐70‐8, Merck Millipore) powders were sieved upon addition to water. The candelilla wax (E902, Koster Keunen Holland BV, The Netherlands) was grated to small pieces (to promote its dissolution) with a sharp knife before addition to olive oil (local supermarket in Wageningen, The Netherlands). Prior to this step, the solvent (Table 1) was stirred on a stirrer plate. As soon as the gelling agent was fully dissolved, the heat stirrer was turned to 50 °C. Upon the gel's formation, the rehydrated BS (see Table 1) were added and mixed by a stirring rod to create a homogeneous mixture. The stirring rod was removed and the mixture was transferred to a silicone baking tray with half‐sphere‐shaped cups (diameter 3 cm, height 1.5 cm). The tray was placed at 4 °C (for no longer that 30 min). The final volumes obtained were divided equally over the number of cups required. The samples were prepared fresh each day of the intervention.

### 2.3 Human Intervention

Five healthy volunteers participated in the in vivo cross‐over study (two males, three females, aged 22–52, Caucasian, healthy BMI). The participants gave written informed consent after being informed about the study. The study was conducted at the Food Quality and Design group at Wageningen University (The Netherlands). The participants consumed the samples in each session at 10:00 a.m. and collected urine in separate pots (urine sampling pots, brown, 500 mL) every bathroom visit during 24 h after consumption of the sample (around every 3 h, except during the night). Prior to consumption, participants had to collect a zero‐measurement urine sample used to check if their diet had been free from other sources of GLs. They marked the urine pots with the time of collection (hh:mm). The participants had a GLs free diet from 48 h before sample consumption, until collection of the last urine sample. At least 2 days of washout occurred between each session. The urine pots were placed at 4 °C directly after sampling and stored for max 24 h. During each session, the participants consumed 0.64 g of BS powder (two gels for the protein and fiber matrix, one gel for the oil sample, one control sample).

### 2.4 Glucoraphanin and Glucoiberin Determination

GLs were determined upon desulfation according to the literature.^7^ Freeze‐dried BS (BS+) (0.1 g DW), microwaved, and freeze‐dried BS (BS−)(0.1 g DW), and in C+ and C− (BS+ and BS− after rehydration) (the whole portion, see Table 1) were extracted with 2.4 mL of methanol (100%) at 75 °C and 200 μL of internal standard solution was added (glucotropaeolin, 3 mm). After incubation for 20 min at 75 °C and centrifugation (1363 × *g*) the supernatant was collected, and the same procedure was repeated another two times on the pellet with 2.4 mL methanol (70%) at 75 °C and the supernatants were combined. The extracted GLs were desulfated according to the literature.^19^ The desulfo‐GLs were separated using a Lichrospher 100 column (Merck RP‐18, 5 μm) with a flow rate of 1 mL min^–1^ (HPLC). The injection volume was 20 μL. Elution was performed using a gradient of water and acetonitrile: A) water and B) acetonitrile, from 0 to 2 min, 0% B; from 2 to 7.5 min, 0–8% B; from 7.5 to 14 min, 8–25% B; from 14 to 18 min, 25% B; from 18 to 20 min, 25–0% B; from 20 to 25 min, 0% B as post‐run. Detection was performed with DAD detector (Spectra System UV 6000 LP) at 229 nm.

### 2.5 Myrosinase Determination

MYR was extracted from BS+ and BS− as described in the literature.^5^ The samples (0.1g DW) were extracted overnight in 70 mL of potassium phosphate buffer (50 mm pH 7.0). Then, the extracts were centrifuged at 2670 × *g* for 10 min and the supernatants were filtrated (folded filters Grade 595 ½—4–7 μm, Whatman) to further clean the solution. The MYR extracts (2 mL) were concentrated using filter centrifugation tubes (Amicon Ultra‐4 cut‐off 30 kD, Millipore, centrifugation at 4000 × *g* for 10 min) to remove compounds with low molecular weight (e.g., sugars and GLs). MYR activity was determined according to a coupled enzymatic procedure described in the literature.^20^ The method is based on the formation of d‐glucose as side product of the reaction between the extracted MYR and a standard sinigrin solution (30 mg mL^–1^). The d‐glucose formed was used to transform NADP+ to NADPH (d‐Glucose assay kit, Megazyme). The formation of NADPH was measured by spectrophotometer (Cary UV 50, Bergen op Zoom, The Netherlands) at 340 nm for 7 min. An external calibration curve was used to quantify MYR activity, by using a standard MYR solution (thioglucosidase, T4528, Sigma‐Aldrich), calibration solutions ranged from 0.02 to 1.2 U mL^–1^. The activity was expressed as U mg^–1^ DW, where one unit MYR enzyme produces 1.0 μmol glucose per minute from sinigrin at pH 6.0 at 25 °C.

### 2.6 Isothiocyanate Determination in the Samples

The ITCs SR and IB were analyzed upon conjugation with 1‐butanethiol.^8^ In brief, 0.32 g DW of BS+, BS−, C+ and C− (*n* = 4), were added to 3 mL of buffer solution (formic acid 0.08 m, triethylamine 15.8 m in methanol) and 300 μL of phenyl ITC solution (20 mm) (internal standard). After 5 min incubation at room temperature, the material was centrifuged at 4415 × *g* for 4 min. The supernatant was collected in another tube and the pellet was extracted again with 3 mL buffer solution and after 5 min shaking and 4 min centrifuging at 4415 × *g* the supernatant was added to the previously collected supernatant. Of this extract, 200 μL was mixed with 12 μL of reagent 1‐butanethiol (99%) and incubated in a heating block at 50 °C for 2 h with occasional shaking of the tube. After that, 4 mL of methanol was added and 1.5 mL of the solution was transferred to the HPLC vial. The conjugates of SR and IB were analyzed using a HPLC equipped with an XBridge RP18 column (3.0 × 100 mm, 5 μm). An injection volume of 10 μL and a flow of 0.4 mL min^–1^ were used. The temperature of the tray was set at 15 °C, and the temperature of the column oven was set at 30 °C. Eluent A was 0.1% formic acid in Millipore water and eluent B was 0.1% formic acid in acetonitrile, the injection volume was 10 μL at 15 °C. The gradient was from 0 to 6 min, 50% B; from 6 to 7.1 min, 95% B; from 7.1 to 10 min, 50% B.

### 2.7 Isothiocyanate Conjugate Determination in Urine

The volume of each urine sample was recorded in order to calculate the cumulative amount of ITC conjugates. For each urine sample, the duplicate of ITC conjugate analysis was performed. The analysis on the ITC conjugates was performed following Vermeulen et al. (2008) with some modifications.^8^ A volume of 200 μL of urine was pipetted into a glass tube with a screw cap. Then, 20 μL of internal standard solution (phenyl isothiocyanate solution, 20 mm), 800 μL of buffer solution (formic acid 0.08 m, triethylamine 15.8 m in methanol), and 12 μL of reagent 1‐butanethiol (99%) were added. Then the tubes were vortexed, placed in the heating block at 50 °C and the same procedure was followed as for the BS samples. The SR and IB conjugates were analyzed using LC‐MS/MS (TSQ Quantum, Thermo Instruments) with an XBridge RP18 column (3.0 × 100 mm, 5 μm). An injection volume of 10 μL and a flow of 0.4 mL min^–1^ were used. The temperature of the tray was 15 °C, of the column oven 30 °C. Eluent A was 0.1% formic acid in Millipore water and eluent B was 0.1% formic acid in acetonitrile, the injection volume was 10 μL at 15 °C. The gradient was from 0 to 6 min, 50% B; from 6 to 7.1 min, 95% B; from 7.1 to 10 min, 50% B. Electrospray ionization in positive mode was used with the following source settings: sheath gas pressure, 45%; auxiliary gas flow, 10%; capillary temperature, 375 °C; source voltage, 3.8 kV. The transitions from the precursor ion to the product ion were as follows: from 254.00 to 164.00 (*m*/*z* values) for SR conjugates, from 268.00 to 178.00 for the iberin conjugates and from 226.00 to 136.00for the internal standard.

### 2.8 Statistical Analysis and Bioavailability Calculations

Bioavailability of SR and IB has been calculated in a different way in the two sets of consumed samples. In the (+) samples bioavailability has been calculated by dividing the cumulative SR or IB amount (μmol) by the initial corresponding ITC content formed upon incubation, before incorporation of BS in the gels (i.e., in C+, see **Table** 2). In the (−) samples, bioavailability has been calculated by dividing the cumulative SR or IB amount (μmol) by the initial content of the corresponding GL before incorporation of BS in the gels (i.e., in C−, see Table 2). Repeated measure ANOVA has been used to determine significant differences among cumulative excretion of SR and IB metabolites in the two sets of gels, with and without active MYR. Significance level was set at 0.05. Sphericity of the data was first checked by means of Mauchly's test (*p* > 0.05 for assumed sphericity). Where sphericity could not be assumed, a Greenhouse–Geisser correction was applied. Post hoc tests with Bonferroni correction were run for the pairwise comparison among the treatments. *T*‐test has been used to determine significant difference between the GLs in BS+ and BS− samples. Results were analyzed by SPSS 23.0 (US).

**Table 2:** Glucoraphanin, glucoiberin, sulforaphane, and iberin content after rehydration in the broccoli sprouts powder with already formed isothiocyanates (C+) and with glucosinolates and inactivated myrosinase (C−). The results are reported per sample given to the study's volunteers

Compound	μmol in 0.64 g of C+	μmol in 0.64 g of C−
	± SD	± SD
Glucoraphanin (GR)	n.d.	10.39 ± 1.48
Glucoiberin (GI)	n.d.	1.30 ± 0.21
Sulforaphane (SR)	5.18 ± 0.66	n.d.
Iberin (IB)	0.56 ± 0.10	n.d.

n.d., not detected.

## 3 Results

### 3.1 Glucosinolates and Isothiocyanates Concentration in the Samples

Pairwise comparison of GR and GI concentration in BS− and BS+ samples before rehydration showed that those concentrations were not significantly different (*p* = 0.86). Therefore, the concentrations of the BS− and BS+ were pooled together (16.1 ± 2.3 μmol g^–1^ DW for GR and 2.03 ± 0.3 μmol g^–1^ DW for GI), whereas SR and IB were not detectable in both the samples. No MYR activity was detected in the microwaved sample whereas MYR activity was 170 ± 17 U mg^–1^ DW in the freeze‐dried samples. The content in GR, GI, and SR, IB after rehydration is reported in Table 2. It can be noticed that approximately half of the initial GR was converted into SR and approximately one third of the initial GI was converted into IB after grinding and rehydration of the BS (+) samples.

### 3.2 Excretion Peak Time (*t*_max_) of Sulforaphane and Iberin

**Figure 2:**
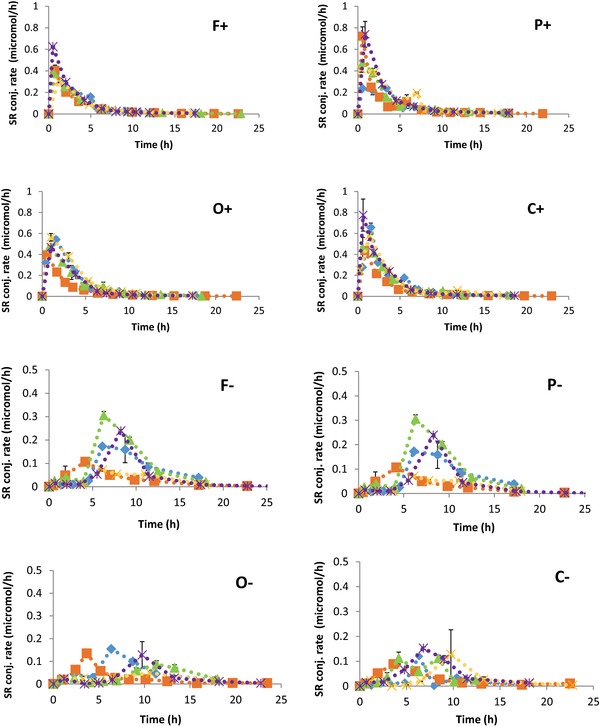
Excretion curves of sulforaphane (SR) conjugates in the timed urine samples during 24 h after consumption of the samples. Different symbols represent different participants (five participants). Proteins (P), dietary fiber (F), and oil (O). Samples with already formed SR (+) and samples with glucoraphanin and inactivated myrosinase (−).

The *t*_max_ was graphically determined from the urinary SR and IB conjugate excretion rates plotted versus time. The characteristic peak time and shape of the excretion curves are represented in **Tables** 3 and 4 and **Figure** 2. Because of the similarity of the excretion curves of SR and IB conjugates, in Figure 2 only the excretion curves of SR conjugates are shown. Such similarity is shown by the *t*_max_ values (Tables 3 and 4). The *t*_max_ values give information on formation of SR and IB and their absorption/excretion after consumption of the samples. The main difference in the *t*_max_ and the shape of the excretion curve can be noticed between the (+) samples and the (−) samples. The SR and IB excretion is delayed by around 6–7 h after consumption of the (−) samples compared to the (+) samples. Little inter‐individual differences were observed among the study participants consuming the same sample.

**Table 3:** Cumulative sulforaphane (SR) conjugates excreted in 24 h urine, SR bioavailability (%), and SR peak time determined in the spot urine samples. Samples served during the intervention, gel type: dietary fiber (F), proteins (P), and oil (O). Control samples (broccoli sprouts) are indicated with (C). Samples with already formed isothiocyanates are indicated by (+) and samples with glucosinolates and inactivated myrosinase are indicated by (−)

Sample	F+	P+	O+	C+	F−	P−	O−	C−
Cumulative SR conjugates excreted in 24 h urine [μmol ± SD]	1.51 ± 0.17	1.88 ± 0.38	1.95 ± 0.52	2.06 ± 0.36	1.2 ± 0.54	1.14 ± 0.50	0.82 ± 0.26	0.66 ± 0.2
SR bioavailability (%) calculated as cumulative SR/GR μmol in before incorporation in the gels	n.a.	n.a.	n.a.	n.a.	11.5 ± 5.2	11.0 ± 4.9	7.9 ± 2.5	6.3 ± 2.0
SR bioavailability (%) calculated as cumulative SR/SR μmol in before incorporation in the gels	29.4 ± 3.3	36.3 ± 7.4	37.6 ± 10.1	39.7 ± 7.0	n.a.	n.a.	n.a.	n.a.
Peak time (*t*_max_, h ± SD) for SR conjugate excretion	1.48 ± 0.51	1.17 ± 0.32	1.82 ± 0.46	1.66 ± 0.46	7.55 ± 1.62	9.32 ± 3.66	9.26 ± 3.69	8.07 ± 2.75

n.a., not applicable.

Different letters within each row of the cumulative IB conjugate values for the samples (for each set of sample (−) and (+)) indicate significant differences (*p* < 0.05). The absence of letters indicates no significant difference.

**Table 4:** Cumulative iberin (IB) conjugates excreted in 24 h urine, IB bioavailability (%), and IB peak time determined in the spot urine samples. Samples served during the intervention, gel type: dietary fiber (F), proteins (P), and oil (O). Control samples (broccoli sprouts) are indicated with (C). Samples with already formed isothiocyanates are indicated by (+) and samples with glucosinolates and inactivated myrosinase are indicated by (−)

Sample	F+	P+	O+	C+	F−	P−	O−	C−
Cumulative IB conjugates excreted in 24 h urine [μmol ± SD]	0.15 ± 0.02ac	0.16 ± 0.05ab	0.21 ± 0.08ac	0.23 ± 0.06c	0.15 ± 0.07	0.14 ± 0.07	0.11 ± 0.03	0.12 ± 0.08
IB bioavailability (%) calculated as cumulative IB/GI μmol in before incorporation in the gels	n.a.	n.a.	n.a.	n.a.	11.8 ± 5.2	10.5 ± 5.3	8.6 ± 2.6	9.2 ± 5.8
IB bioavailability (%) calculated as cumulative IB/IB μmol in before incorporation in the gels	27.2 ± 3.9	28.6 ± 8.3	38.2 ± 14.0	41.1 ± 11.6	n.a.	n.a.	n.a.	n.a.
Peak time (*t*_max_, h ± SD) for IB conjugate excretion	1.48 ± 0.51	2.28 ± 1.43	1.82 ± 0.46	1.94 ± 0.52	8.06 ± 2.12	9.52 ± 3.51	9.26 ± 3.69	8.07 ± 2.75

n.a., not applicable.

Different letters within each row of the cumulative IB conjugates values for the samples (for each set of sample (−) and (+)) indicate significant differences (*p* < 0.05). The absence of letters indicates no significant difference.

### 3.3 Bioavailability

In this study, the results of the cumulative SR and IB conjugates excreted in 24 h urine and the potential bioavailability of SR and IB are shown in Tables 3 and 4. The cumulative amount corresponds to the total SR and IB conjugates (μmol) excreted during the 24 h (timed urine samples) after consumption of the samples. For both SR and IB, the highest values of cumulative excretion in urine are given by all samples with preformed ITC (+) and the control samples (C+) showing the highest values (2.06 ± 0.36 μmol for SR and 0.23 ± 0.06 μmol for IB). The lowest values of the cumulative SR and IB excretion were found in the control samples with GLs containing inactivated MYR (C−) (0.66 ± 0.2 μmol for SR and 0.12 ± 0.08 μmol for IB), which are around three times lower for SR and two times lower for IB than in C+ urine samples.

In (+) samples, the highest bioavailability was observed in the C+ for both SR (39.7 ± 7.0%) and IB (41.1 ± 11.6%), whereas the bioavailability from gels was lower, particularly that of the F+ sample (1.4 times lower for SR and 1.5 times for IB) compared to the C+ sample. However, only the IB bioavailability in the P+ gel was significantly lower (*p* = 0.007). Instead, an opposite trend was observed for the (−) samples: the control C− samples showed the lowest bioavailability for both conjugates, whereas the F− samples had the highest values (1.8 times for SR and 1.2 times for IB higher). The consumption of P− samples showed a bioavailability higher than the C− samples as well (1.7 times for SR and 1.2 for IB higher).

## 4 Discussion

### 4.1 Gel Preparation

The aim of this study was to explore the influence of broccoli co‐digestion with single food ingredients protein, oil, and dietary fiber on the bioavailability of SR and IB and the excretion rates of their conjugates. Food biopolymers and macromolecules can occur in foods in different forms/structures, for instance as emulsions or networks, which may differentially affect the colloidal and rheological properties of digesta and thus bioavailability of bioactive compounds. To eliminate such effects, it was chosen to deliver the food biopolymer in one type, that is, incorporating BS in a gel. The gelling conditions have been optimized to obtain gels that could be served to the participants in the morning, and that could preserve ITCs and GLs during the incorporation of the two types of BS samples, (+) and (−), into the gels (e.g., fast gel preparation at temperature not higher than 50 °C).

In the first phase of the study, standardized procedures were developed to 1) obtain BS with active MYR, and BS with inactive MYR, and 2) to rehydrate the BS with active MYR in order to obtain a controlled SR and IB formation. The inactivation of MYR was successful (Table 2), since no residual MYR activity could be detected by the spectrophotometric assay which was confirmed by the lack of SR and IB formation during rehydration of the BS− material. Those results are in line with previous studies, although in those studies different *Brassica oleracea* varieties were used.^4^, ^7^, ^19^, ^21^ The controlled formation of SR and IB was also successfully standardized as reported in Table 2, in which the concentration of SR and IB in four independently rehydrated BS+ samples (from the same BS+ batch) is reported with a relative standard deviation of 12% for SR and 18% for IB. Moreover, when adding the incubated BS into the gels, the gel temperature did not exceed 50 °C, to prevent thermal degradation of GLs and ITCs.^21^, ^22^ The standardization of the rehydration procedure is crucial for the correct interpretation of quantitative data from human intervention studies where the functional properties of ITCs are investigated.

The concentration of GR and GI in BS (16.1 ± 2.3 μmol g^–1^ DW for GR and 2.0 ± 0.3 μmol g^–1^ DW for GI) is similar to the concentration of other broccoli sprouts reported in the literature (14.6 ± 3.1 μmol DW^–1^ for GR and 6.6 ± 1.2 μmol DW^–1^ for GI).^23^

### 4.2 Excretion of Peak Time (*t*_max_) of Sulforaphane and Iberin and Excretion Curves

The shape of the excretion curves and the *t*_max_ of SR and IB are very similar within either the (+) samples or (−) samples, suggesting a very similar pharmacokinetics for the two ITCs. This result was expected since SR and IB differ by only one methyl group in the side chain and similar results were obtained in other studies.^24^ A clear difference could be noticed comparing the profiles of the (+) samples and (−) samples. In the latter, the *t*_max_ of ITCs which are produced by the gut microbiota was delayed by 6–7 h compared to the *t*_max_ of the preformed ITC in the (+) samples. These differences in time are caused by a difference in the absorption site: the preformed ITCs can be absorbed by the small intestine, while the intact GLs first have to be converted into their ITC by MYR‐like activity of our gut microbiota in the colon. The ability of the gut microbiota to convert GL into breakdown products, including ITCs, depends on the microbiota profile.^7^, ^25^, ^26^ On the contrary, the *t*_max_ of SR and IB after consumption of the (+) samples was just after 1.17 ± 0.32 and 1.94 ± 0.52 h, in line with previously published studies.^7^, ^25^, ^26^ These values suggest a very fast absorption of preformed ITCs or ITCs formed from the GL precursors during chewing broccoli with active MYR.

### 4.3 SR and IB Bioavailability

The estimation of the potential bioavailability of SR and IB was carried out by three calculations: the cumulative amount of the conjugated SR and IB excreted in urine, the bioavailability relative to the corresponding GL precursor (for the (−) samples) and the bioavailability relative to the ITC (for the (+) samples). As a general observation, the standard deviation of the bioavailability in the (−) samples is higher than standard deviation of the bioavailability in the (+) samples (between 11% and 38% for (+) samples and 27% and 66% for (−) samples). Such high standard deviation suggests that the inter‐variability among the participants in the excretion of SR and IB is higher after consumption of (−) samples compared to when consuming (+) samples. Such variability is confirmed by other studies^7^, ^27^ and it can be explained by differences in microbiota population in different individuals. This interindividual variability in the conversion rate may depend on the specific β‐thioglucosidase activity of the gut microbiota, but also to interconversion between breakdown products and/or further metabolism of the ITC by the gut microbiota (and from different physiological conditions occurring in the large intestine). Recently, it has been shown that the hydrolysis yield may increase by exposure of the gut microbiota to GLs, that is, by consumption of *Brassica* vegetables.^28^ The variability in SR and IB bioavailability in the (+) samples can be explained by individual differences in the absorption yield from the intestinal epithelium and in individual differences in the interconversion of SR into erucin.^29^ The gel composition was designed in order to minimize differences deriving from different oral processing behavior, by using gels of similar consistency and by standardizing BS particle size at <450 μm.

In general, the cumulative excretion of SR and IB conjugates was higher after consumption of the (+) samples. SR and IB were also more bioavailable in the (+) samples rather than in the (−) samples. Bioavailability of ITCs ranged between 27% and 41% in the (+) samples and between 6% and 12% in the (−) samples. These results were expected and are confirmed by other reports.^7^, ^8^, ^25^, ^26^, ^27^ The higher bioavailability in the (+) samples is explained by the readily available ITCs that were already hydrolyzed by endogenous still active MYR. Whereas the conversion in ITC after consumption of the (−) samples was catalyzed by the gut microbiota. Therefore, the comparison of the ITCs bioavailability in the (+) and the (−) samples is affected by 1) the bioavailability calculation (different denominators) and by 2) the ITC conversion step that occurs in the gut after consumption of the (−) samples.

It is important to note however that in our study, the bioavailability was calculated by measuring the ITC conjugates in urine after reaction with 1‐butanethiol and this method may not be able to detect all the mercapturic acid derivatives in urine. In a study in which the same method was applied,^7^ an SR bioavailability of 58% and 10% was measured after consumption of broccoli powder with or without active MYR, respectively, compared to a bioavailability of 39.7% in the C+ samples and of 6.3% for the C− samples reported in the present study. The bioavailability measured in these two studies are probably underestimated when compared to bioavailability values from a similarly accessible matrix reported in other studies which make use of the cyclocondensation assay, that is, reaction with 1,2‐benzenedithiol (average bioavailability of 71% in SR‐containing pills),^30^ or that quantified individual mercapturic acids (bioavailability of almost 100% of preformed ITC in condiments).^27^

The main aim of this study was to explore the effect of dietary macromolecules on the bioavailability of ITCs. In this respect an interesting trend can be noticed by looking at the bioavailability within each sample group. The potential bioavailability of ITCs was higher after consumption of the control sample C+ compared to the gels (P+, O+, F +) (Tables 3 and 4). Going more into details, the bioavailability of ITC from the control C+ was 1.4‐fold higher than from the F+ gels. However, for both the ITCs no significant differences were found, but the pairwise comparison showed a *p* = 0.051 between F+ and C+ for SR, which suggests a trend toward a lower bioavailability for ITCs in BS with active MYR incorporated into gels. This effect of fibers on reduction of ITC bioavailability might be explained by binding of ITCs to the hydroxyl groups of alginate or by physical entrapment into the gel network. Alginate is a linear copolymer of α‐*l* ***‐***guluronate and β‐*d*‐mannuronate. Alginate hydrogels have been proposed as controlled delivery systems in the gastrointestinal tract or to control nutrient digestibility.^31^ In general, dietary fiber coatings are a suitable strategy to modulate the release of target nutrients in the gastrointestinal tract, or the accessibility of digestive enzymes to the respective substrates. However, this capacity strongly depends on the internal structure of the alginate gels, particularly the pore size of the gel network of aggregated alginate molecules. The pH changes during digestion also affect the gel structure. At low pH (gastric phase) a potential shrinking of alginate can occur,^32^ and the surface of the alginate gel is converted into a porous, insoluble so‐called alginic acid skin. At higher pH (intestinal phase), the alginic acid skin is converted to a soluble viscous layer.^32^ Moreover, theoretically, alginate gels could have been hydrolyzed at the gut level, by the microbiota, allowing the release of ITCs. However, only specific bacteria can utilize the alginate, for example, bacteria that produce alginate lyase enzymes,^33^, ^34^ potentially releasing the still encapsulated ITCs.

Bioavailability of SR and IB from the P+ sample was lower than in the control C+ sample. However, the pairwise comparison showed this difference to be only significant for IB (*p* = 0.007) but not for SR (*p* = 0.068). ITCs are strongly electrophilic compounds able to react with amino acids containing thiol, hydroxyl, and amino groups.^35^, ^36^ The thiol group is the preferred reaction site for ITCs, whereas amino groups reacted more slowly and only under basic conditions. It was therefore assumed that a limited binding of SR and IB has occurred with nucleophilic groups of gelatine due to the low content in sulfur amino acids and lysine.^37^ In a recent study, the amount of SR recovered after in vitro digestion of broccoli powder co‐digested with bovine serum albumin was not different from the broccoli powder alone.^38^ Gelatine is not particularly resistant to digestion, still the persistence of the protein gel in the gastric phase may have slowed down the release and the absorption of SR and IB from the P+ gels.

One of our preliminary hypotheses was that the O+ gel would lead to higher bioavailability of SR and IB.^14^, ^15^, ^16^, ^17^ This hypothesis could not be confirmed in the present study. The explanation for the higher bioavailability of GL in the presence of a fatty meal is that the presence of lipids would improve micellarization of fat and therefore absorption of lipophilic ITCs. However, in the present study the amount of fat incorporated in the oleogel was probably too low to significantly impact on lipid micellarization. On the other hand, the addition of wax to oil leads to the formation of a 3D network that entraps oil within its pores and by adsorbing oil onto the surface of the network^39^; though, the present results show that the oleogel structure would not represent a significant barrier for the release of micronutrients. However, it must be stressed that oleogels may exhibit a large variety of physico‐chemical properties depending on the type of oil and gelator used and depending on the conditions of heating, shearing, and cooling used for the oleogelation which means that our results may not be necessarily generalizable to all the oleogels.^39^

An opposite trend was observed after the consumption of the (−) samples. The bioavailability after consumption of the control C−was lower than after consumption of F− (1.8‐fold for SR and 1.3‐fold for IB higher) even though the difference was found to be not statistically significant for neither of the two ITCs. Moreover, the consumption of P− also led to a higher bioavailability than after consumption of C−. In the (−) samples, bioavailability mostly depends on the conversion yield of GLs to the corresponding ITC by the gut microbiota, that is, how much GL survive the passage thorough the upper gut. Such lower hydrolysis extent is mainly explained by a less efficient MYR‐like activity of the microbiota^40^, ^41^ and by the loss of GLs during the digestion (thus less substrate for the microbiota), for example, low pH condition in the stomach phase may hydrolase GLs.^26^, ^42^ Despite data from in vitro models must be interpreted with caution in the case of GL, contradictory results have been reported on the stability of GL in the gastric acidic environment.^38^, ^43^ It can be hypothesized that incorporation of BS into a gel may have partly protected the GLs digestion, increasing the amount of GR and GI that reached the gut microbiota intact.

In this study, the effect of mono gels made of proteins, fiber, or fat on the bioavailability and excretion profile of the health‐beneficial SR and IB has been explored.

Five participants took part in this cross‐over study and collected a timed urine sample before consumption of the gels and 24 h timed urine samples after consumption, and the SR and ITC conjugates were determined in the timed urine samples. Samples in which SR and IB were already formed led to a higher bioavailability of those compounds. The fiber‐gel samples led to lower potential bioavailability for the gels rich in SR and IB whereas for the gels rich in their precursors, GR and GI, the opposite trend was observed. These results suggest that the bioavailability of SR and IB may be hindered by the soluble fiber‐gel structure; whereas, GR and GI entrapped in such soluble gel structure may be better preserved until they reach the gut microbiota, increasing the formation of SR and IB. This preliminary study has yielded interesting results that can be used for further investigation on the effects of food matrix structure and composition. In particular, further investigations should focus on the effect on GLs and ITC bioavailability of different dietary fibers and proteins from different sources as well as different structures, recruiting a higher number of participants. Such investigations will give insights to develop food products that lead to an optimized ITC bioavailability.

**Abbreviations**
−: sample containing glucosinolates in which myrosinase was inactivated
+: sample containing isothiocyanates in which myrosinase was still active
BS: broccoli sprouts powder
C: control sample
F: dietary fiber
GI: glucoiberin
GL: glucosinolate
GR: glucoraphanin
IB: iberin
ITC: isothiocyanate
MYR: myrosinase
O: oil
P: proteins
SR: sulforaphane

## Conflict of Interest

The authors declare no conflict of interest.

## References

[1] M. Traka , R. Mithen , Phytochem. Rev. 2009, 8, 269.

[2] R. Kissen , J. T. Rossiter , A. M. Bones , Phytochem. Rev. 2008, 8, 69.

[3] E. Capuano , M. Dekker , R. Verkerk , T. Oliviero , Curr. Pharm. Des. 2017, 23, 2697 28117016 10.2174/1381612823666170120160832

[4] R. Verkerk , M. Dekker , J. Agric. Food Chem. 2004, 52, 7318. 15563214 10.1021/jf0493268

[5] T. Oliviero , R. Verkerk , M. A. J. S. Van Boekel , M. Dekker , Food Chem. 2014, 163, 197. 24912716 10.1016/j.foodchem.2014.04.099

[6] J. W. Fahey , S. L. Wehage , W. D. Holtzclaw , T. W. Kensler , P. A. Egner , T. A. Shapiro , P. Talalay , Cancer Prev. Res. 2012, 5, 603. 10.1158/1940-6207.CAPR-11-0538 PMC4618677 22318753

[7] T. Oliviero , R. Verkerk , M. Vermeulen , M. Dekker , Mol. Nutr. Food Res. 2014, 58, 1447. 24687744 10.1002/mnfr.201300894

[8] M. Vermeulen , I. W. A. A. Klöpping‐Ketelaars , R. van den Berg , W. H. J. Vaes , J. Agric. Food Chem. 2008, 56, 10505. 18950181 10.1021/jf801989e

[9] N. V. Matusheski , J. A. Juvik , E. H. Jeffery , Phytochem. 2004, 65, 1273. 10.1016/j.phytochem.2004.04.013 15184012

[10] F. S. Hanschen , R. Klopsch , T. Oliviero , M. Schreiner , R. Verkerk , M. Dekker , Sci. Rep. 2017, 7, 40807. 28094342 10.1038/srep40807 PMC5240131

[11] L. Ye , A. T. Dinkova‐Kostova , K. L. Wade , Y. Zhang , T. A. Shapiro , P. Talalay , Clin. Chim. Acta 2002, 316, 43. 11750273 10.1016/s0009-8981(01)00727-6

[12] T. A. Shapiro , J. W. Fahey , K. L. Wade , K. K. Stephenson , P. Talalay , Cancer Epidemiol. Biomarkers Prev. 2001, 10, 501. 11352861

[13] J. Kroll , H. Rawel , R. Kröck , J. Proll , W. Schnaak , Mol. Nutr. Food Res. 1994, 38, 53.

[14] V. Rungapamestry , A. J. Duncan , Z. Fuller , B. Ratcliffe , Br. J. Nutr. 2007, 97, 644. 17349076 10.1017/S0007114507381403

[15] A. J. Humberstone , W. N. Charman , Adv. Drug Deliv. Rev. 1997, 25, 103.

[16] K. Ippoushi , H. Ueda , A. Takeuchi , Food Chem. 2014, 161, 176. 24837937 10.1016/j.foodchem.2014.04.013

[17] K. Ippoushi , H. Ueda , A. Takeuchi , Food Chem. 2013, 141, 1192. 23790903 10.1016/j.foodchem.2013.03.058

[18] N. C. Howarth , E. Saltzman , S. B. Roberts , Nutr. Rev. 2001, 59, 129. 11396693 10.1111/j.1753-4887.2001.tb07001.x

[19] K. Oerlemans , D. M. Barrett , C. B. Suades , R. Verkerk , M. Dekker , Food Chem. 2006, 95, 19.

[20] D. Van Eylen , M. Hendrickx , A. Van Loey , Food Chem. 2006, 97, 263.

[21] T. Oliviero , R. Verkerk , M. Dekker , Food Chem. 2012, 132, 2037. 10.1016/j.foodchem.2014.04.099 24912716

[22] D. Van Eylen , I. Oey , M. Hendrickx , A. Van Loey , J. Agric. Food Chem. 2007, 55, 2163. 17305356 10.1021/jf062630b

[23] Q. Tian , R. A. Rosselot , S. J. Schwartz , Anal. Biochem. 2005, 343, 93. 15963940 10.1016/j.ab.2005.04.045

[24] T. Pilipczuk , B. Kusznierewicz , T. Chmiel , W. Przychodzeń , A. Bartoszek , Food Chem. 2017, 214, 587. 27507514 10.1016/j.foodchem.2016.07.125

[25] J. W. Fahey , W. D. Holtzclaw , S. L. Wehage , K. L. Wade , K. K. Stephenson , P. Talalay , PloS One 2015, 10, e0140963. 26524341 10.1371/journal.pone.0140963 PMC4629881

[26] D. Angelino , E. Jeffery , J. Funct. Foods 2014, 7, 67.

[27] M. Vermeulen , R. Van den Berg , A. P. Freidig , P. J. Van Bladeren , W. H. Vaes , J. Agric. Food Chem. 2006, 54, 5350. 16848516 10.1021/jf060723n

[28] F. Li , M. A. Hullar , S. A. Beresford , J. W. Lampe , FASEB J. 2010, 24, lb371.

[29] J. D. Clarke , A. Hsu , K. Riedl , D. Bella , S. J. Schwartz , E. Ho , Pharmacol. Res. 2011, 64, 456. 21816223 10.1016/j.phrs.2011.07.005 PMC3183106

[30] J. W. Fahey , K. L. Wade , S. L. Wehage , W. D. Holtzclaw , H. Liu , P. Talalay , E. Fuchs , K. K. Stephenson , Mol. Nutr. Food Res. 2017, 61, XX. 10.1002/mnfr.201600766 27935214

[31] M. N. Corstens , C. C. Berton‐Carabin , P. T. Elichiry‐Ortiz , K. Hol , F. J. Troost , A. A. M. Masclee , J. Funct. Foods 2017, 34, 319.

[32] M. George , T. E. Abraham , J. Control. Release 2006, 114, 1. 16828914 10.1016/j.jconrel.2006.04.017

[33] M. Li , G. Li , Q. Shang , X. Chen , W. P. X. Liu , L. Zhu , Y. Yin , G. Yu , X. Wang , Anaerobe 2016, 39, 19. 26891629 10.1016/j.anaerobe.2016.02.003

[34] S. Bai , H. Chen , L. Zhu , W. Liu , H. D. Yu , X. Wang , Y. Yin , PloS One 2017, 12, e0171576. 28170428 10.1371/journal.pone.0171576 PMC5295698

[35] Y. Zhang , P. Talalay , Cancer Res. 1994, 54, 1976s. 8137323

[36] M. Hernández‐Triana , J. Kroll , J. Proll , J. Noack , K. J. Petzke , J. Nutr. Biochem. 1996, 7, 322.

[37] C. T. Hue , N. T. M. Hang , R. Razumovskaya , Turk. J. Fish Aquat. Sci. 2017, 17, 1117.

[38] I. Sarvan , E. Kramer , H. Bouwmeester , M. Dekker , R. Verkerk , Food Chem. 2017, 214, 580. 27507513 10.1016/j.foodchem.2016.07.111

[39] F. C. Wang , A. J. Gravelle , A. I. Blake , A. G. Marangoni , Curr. Opin. Food Sci. 2016, 7, 27.

[40] R.‐H. Lai , M. J. Miller , E. Jeffery , Food Funct. 2010, 1, 161. 21776467 10.1039/c0fo00110d

[41] T. A. Shapiro , J. W. Fahey , K. L. Wade , K. K. Stephenson , P. Talalay , Cancer Epidemiol. Biomarkers Prev. 1998, 7, 1091. 9865427

[42] I. Maskell , R. Smithard , Br. J. Nutr. 1994, 72, 455. 7524654 10.1079/bjn19940047

[43] F. Vallejo , A. Gil‐Izquierdo , A. Pérez‐Vicente , C. García‐Viguera , J. Agric. Food Chem. 2004, 52, 135. 14709026 10.1021/jf0305128

